# Albany Medical College

**Published:** 1870-04

**Authors:** 


### Albany Medical College.

Dr. Thomas C. Durant, of New York, a graduate of the college and an early student of Drs. March and Armsby, has given $15,000 to endow the "March Professorship."

Drs. E. R. Peaslee and Meredith Clymer, of New York, and Dr. Wm. P. Seymour, of Troy, have accepted chairs in the new Faculty.

